# Undifferentiated prostate cancer treated with docetaxel

**DOI:** 10.1002/iju5.12162

**Published:** 2020-04-20

**Authors:** Masaki Kobayashi, Minato Yokoyama, Masaharu Inoue, Yasuhisa Fujii

**Affiliations:** ^1^ Department of Urology Tokyo Medical and Dental University Tokyo Japan

**Keywords:** chemotherapy, docetaxel, prostate cancer, quality of life, undifferentiated carcinoma

## Abstract

**Introduction:**

Undifferentiated prostate cancer is a rare and aggressive disease, and no definitive therapeutic strategy for metastatic undifferentiated prostate cancer has been established.

**Case presentation:**

We report the case of a 70‐year‐old man with undifferentiated prostate cancer and lymph node metastases. Following the patient’s progression after platinum‐based chemotherapy, we initiated treatment with docetaxel, which resulted in a partial response. The patient was alive for 42 months after initiation of docetaxel, maintaining relatively good quality of life with minimal hospital stay.

**Conclusion:**

Docetaxel may be a good therapeutic option for metastatic undifferentiated prostate cancer.

**Abbreviations & Acronyms**
CT: computed tomography
EBRT: external beam radiotherapy
EP: etoposide and cisplatin
FDG‐PET: fluorodeoxyglucose positron emission tomography
PSA: prostate‐specific antigen
SOX: S‐1 and oxaliplatin

**Keynote message**

We reported a case of metastatic undifferentiated prostate cancer treated with docetaxel. Undifferentiated prostate cancer is a rare and aggressive disease, and there is no established therapeutic strategy. This case indicated that docetaxel might be a good option for metastatic undifferentiated prostate cancer.

## Introduction

Undifferentiated carcinoma of the prostate is a rare tumor, comprising 0.5% of all malignant tumors of the prostate.^1^ No definitive treatment strategy for metastatic undifferentiated prostate cancer has been established, and its prognosis is very poor.^2^

Although docetaxel is a key agent for castration‐resistant prostate cancer,^3^, ^4^ its efficacy in undifferentiated prostate cancer is unknown. Several reports showed favorable outcomes in undifferentiated stomach or thyroid cancer treated with docetaxel,^5^, ^6^, ^7^ which suggested that docetaxel could be a preferable option for undifferentiated prostate cancer.

Herein, we report a case of metastatic undifferentiated prostate cancer in which docetaxel achieved a favorable clinical response and long‐term survival.

## Case presentation

**Fig. 1:**
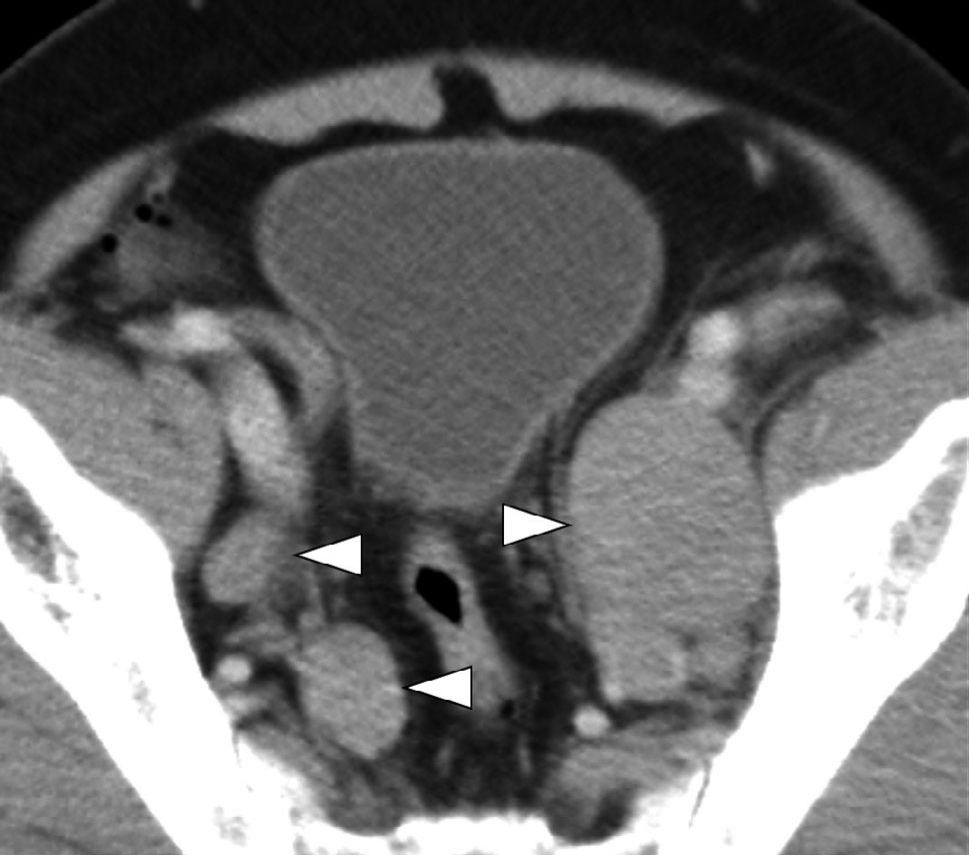
CT image at presentation showing multiple swollen pelvic lymph nodes (arrowheads).

A 70‐year‐old Japanese man presented with lower abdominal pain in March 2009. CT showed multiple swollen lymph nodes in the retroperitoneum and pelvis (Fig. 1). Serum tumor markers, including carcinoembryonic antigen, carbohydrate antigen 19‐9, soluble interleukin‐2 receptor, neuron‐specific enolase, and progastrin‐releasing peptide, were within normal limits. PSA of 1.29 ng/mL was also within normal limit.

**Fig. 2:**
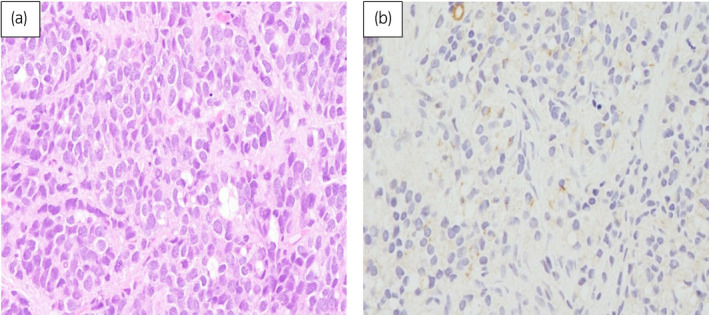
Microscopic features of the prostate biopsy specimens (hematoxylin and eosin staining). The specimens were occupied by tumor cells which had large round nuclei with prominent nucleoli and proliferated in a sheet‐like pattern (a), and weakly positive for PSA staining (b).

Open biopsy of the para‐aortic lymph nodes revealed that the lymph nodes were occupied with undifferentiated malignant cells. Immunohistochemically, the tumor cells were positive for pan‐cytokeratin and weakly positive for chromogranin A and synaptophysin, but negative for AE1/AE3, CK7, CK20, CD3, CD20, CD79a, and PSA. Although the patient underwent intensive examinations, such as upper and lower gastrointestinal endoscopy and ^18^F‐FDG‐PET/CT, the origin of the cancer could not be identified. In July 2009, suspected of having occult primary digestive tract cancer,^8^ the patient received S‐1 40 mg/m^2^ on days 1–14 and oxaliplatin 100 mg/m^2^ on day 1 (SOX therapy). After one course of SOX therapy, the maximum diameter of the prostate showed enlargement from 56 to 61 mm in addition to increasing size of pelvic lymph nodes. In addition, FDG‐PET/CT showed the uptake in the prostate in a retrospective view. Therefore, prostate biopsy was performed and it revealed undifferentiated carcinoma, showing similar characteristics as the para‐aortic lymph node cells, except for weakly positive PSA staining (Fig. 2). On the basis of these findings, the patient was diagnosed with undifferentiated prostate cancer, cT3aN1M1a.

Because the weakly positive staining for chromogranin A and synaptophysin suggested neuroendocrine differentiation, etoposide 100 mg/m^2^ on days 1–5 and cisplatin 20 mg/m^2^ on days 1–5 (EP therapy) were introduced as a second‐line systemic treatment. Consequently, grade 4 neutropenia and thrombocytopenia were observed for 7 days, and urinary retention and edema of the lower extremities occurred due to enlargement of the prostate and pelvic lymph nodes. The patient underwent EBRT to the whole pelvis at 44 Grays, combined with androgen blockade, followed by EBRT to the para‐aortic area at the same dose. Although the lymph nodes shrank and the symptoms disappeared, the pelvic and para‐aortic lymph nodes again became large and left supraclavicular lymph node metastases developed 10 months after initial EBRT.

**Fig. 3:**
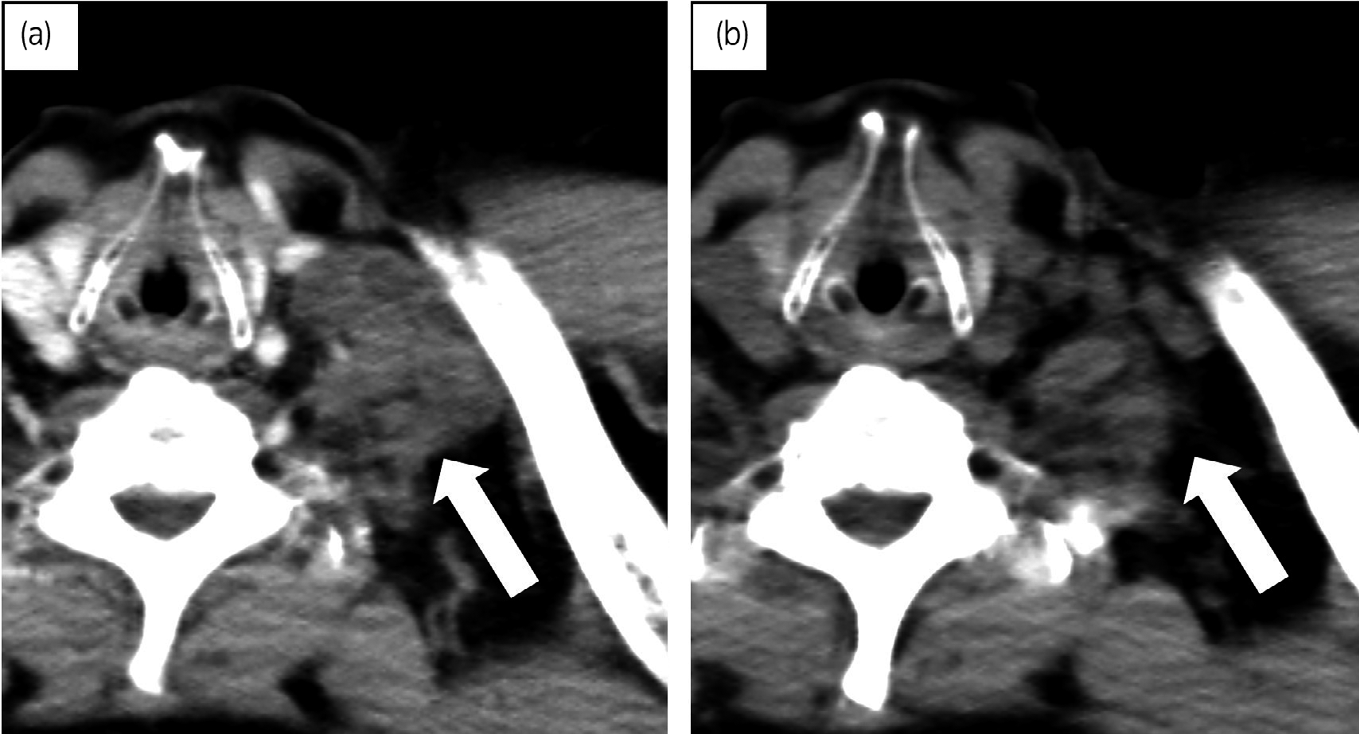
CT images before initiation of docetaxel (a) and after two courses of docetaxel (b) showing remarkable shrinkage of swollen left supraclavicular lymph nodes (arrow).

In September 2011, docetaxel therapy was started. For prevention of the severe myelosuppression and because of the patient's wish to maintain his quality of life, docetaxel was administered at a relatively low dose intensity of 50 mg/m^2^ at 4‐week intervals. After two initial cycles of docetaxel, partial response was achieved without major adverse events (Fig. 3). The patient complained of peripheral neuropathy requiring discontinuation of chemotherapy after the ninth course of docetaxel. Four months later, the pelvic lymph nodes again showed enlargement. Since the patient’s peripheral neuropathy lessened, docetaxel was re‐started at the same dose every 6 weeks. Although temporary stable disease was achieved for 5 months, multiple bone metastases were found after the 14th course. The patient ultimately died of the disease in March 2015 at 19 months after withdrawal of docetaxel.

## Discussion

We reported a case of undifferentiated prostate cancer with lymph node metastases treated with docetaxel, which resulted in a partial response and long‐term survival.

Because the standard treatment for undifferentiated prostate cancer has not been established due to its rarity, several treatments have been introduced. However, their outcomes were not satisfactory. The median time of overall survival ranged from 12 to 18 months, which was clearly shorter than the median survival typically observed in more common types of prostate cancer.^2^, ^9^, ^10^ Consistent with these earlier reports, platinum‐based chemotherapy, such as SOX or EP therapy, did not prevent disease progression in our case. Although chemoradiotherapy combined with hormonal therapy played a role in shrinking the tumor and controlling some symptoms caused by cancer progression, its efficacy was limited to temporary local control.

There are some reports on the efficacy of docetaxel for the treatment of undifferentiated carcinoma of stomach and thyroid gland. Kawada *et al*. reported on a prospective study of seven patients with undifferentiated thyroid cancer treated with docetaxel administered every 3 weeks. Of the seven patients, one achieved a complete response and two maintained stable disease.^5^ High efficacy of docetaxel combined with radiation was also demonstrated for undifferentiated thyroid cancer.^6^ Furthermore, docetaxel combined with S‐1 was reported to diminish liver metastasis in a case of undifferentiated gastric cancer.^7^

To our knowledge, there are no previous reports on the use of docetaxel for the treatment of undifferentiated prostate cancer. However, it is feasible that docetaxel could have efficacy in undifferentiated prostate cancer. In fact, docetaxel prevented disease progression in our case for 20 months in total (partial response was maintained for 15 months, and stable disease for 5 months), and the patient survived for 42 months from initiation of docetaxel. One possible explanation why docetaxel was effective for undifferentiated prostate cancer was that the tumor might retain some characteristics of prostatic adenocarcinoma because PSA staining was positive in a portion of the tumor cells. Distinction between undifferentiated carcinoma and poorly differentiated adenocarcinoma would not be necessarily easy due to heterogeneity and seamless transition of prostate cancer, because the histological diagnosis is usually based on a small amount of tissue samples in clinical settings. Therefore, a non‐negligible proportion of poorly differentiated adenocarcinoma of the prostate might be overlooked.

In the present case, relative dose intensity of docetaxel was reduced compared with that used for castration‐resistant prostate cancer to maintain the patient’s quality of life. Docetaxel therapy was continued without requiring hospitalization, and except for the reversible peripheral neuropathy, no other adverse events interfered with the planned treatment.

We reported a case of metastatic undifferentiated prostate cancer in which docetaxel achieved a favorable clinical response and long‐term survival. Docetaxel may be a good therapeutic option for undifferentiated prostate cancer.

## Conflict of interest

The authors declare no conflict of interest.

## References

[1] Helpap B , Köllermann J . Undifferentiated carcinoma of the prostate with small cell features: immunohistochemical subtyping and reflections on histogenesis. Virchows Arch. 1999; 434: 385–91. 10389621 10.1007/s004280050357

[2] Hashimoto K , Sasajima Y , Ando M *et al* Immunohistochemical profile for unknown primary adenocarcinoma. PLoS One 2012; 7: e31181. 22299055 10.1371/journal.pone.0031181 PMC3267772

[3] Tannock IF , de Wit R , Berry WR *et al* Docetaxel plus prednisone or mitoxantrone plus prednisone for advanced prostate cancer. N. Engl. J. Med. 2004; 351: 1502–12. 15470213 10.1056/NEJMoa040720

[4] Petrylak DP , Tangen CM , Hussain MH *et al* Docetaxel and estramustine compared with mitoxantrone and prednisone for advanced refractory prostate cancer. N. Engl. J. Med. 2004; 351: 1513–20. 15470214 10.1056/NEJMoa041318

[5] Kawada K , Kitagawa K , Kamei S *et al* The feasibility study of docetaxel in patients with anaplastic thyroid cancer. Jpn. J. Clin. Oncol. 2010; 40: 596–9. 20200039 10.1093/jjco/hyq025

[6] Troch M , Koperek O , Scheuba C *et al* High efficacy of concomitant treatment of undifferentiated (anaplastic) thyroid cancer with radiation and docetaxel. J. Clin. Endocrinol. Metab. 2010; 95: E54–E57. 20591979 10.1210/jc.2009-2827

[7] Kitahara H , Oki E , Saeki H *et al* A case of liver metastasis from gastric cancer responding completely to S‐1/docetaxel chemotherapy. Gan To Kagaku Ryoho. 2013; 40: 1093–7. 23986059

[8] Hong YS , Park YS , Lim HY *et al* S‐1 plus oxaliplatin versus capecitabine plus oxaliplatin for first line treatment of patients with metastatic colorectal cancer: a randomized, non‐inferiority phase 3 trial. Lancet Oncol. 2012; 13: 1125–32. 23062232 10.1016/S1470-2045(12)70363-7

[9] D’Aprile M , Santini D , Di Cosimo S *et al* Atypical case of metastatic undifferentiated prostate carcinoma in a 36 years old man: clinical report and literature review. Clin. Ter. 2000; 151: 371–4. 11141722

[10] Demirci U , Coskun U , Karaka H *et al* Docetaxel and cisplatin in first line treatment of patients with unknown primary cancer: a multicenter study of the Anatolian Society of Medical Oncology. Asian Pac. J. Cancer Prev. 2014; 15: 1581–4. 24641371 10.7314/apjcp.2014.15.4.1581

